# A Valuable Report to the Medical Research Council

**Published:** 1921-03-05

**Authors:** 


### AMŒBIC DYSENTERY IN BRITAIN.

### A Valuable Report to the Medical Research Council.

The publication of Mr. Clifford Dobell's work upon the occurrence of intestinal protozoa among the inhabitants of this country has provided all health workers with some much needed technical information. Prior to the war practically nothing was certainly known on this matter. Diseases due to intestinal protozoa were not generally recognised as occurring in the British Isles. The majority of medical men regarded them as peculiar to the tropics and left the matter in the hands of physicians practising in that sphere.

Within recent years certain outstanding points have been widely appreciated, such as the risk from soldiers infected with the *Entamœba histolytica* (the dysentery amœba), and the fact that healthy human carriers of this parasite are not uncommon. But, until now, no reliable information has been to hand of the number of people who have never left these shores but who are, nevertheless, definitely infected.

### Home-contracted Dysentery.

That such people can and do contract active amœbic dysentery is now certain. Many instances have been observed, but the first of these only need be noted. In 1916, Dr. C. M. Wenyon studied the case of a labourer in London who had never been out of England but had worked on a transport lying in dock. In the London hospital he developed typical amœbic dysentery, including liver abscess and intestinal ulceration; the typical protozoa were recovered in laboratory examinations. A large number of other examples followed, and later on, as the systematic examination of all troops became more general, such a very large number of "carriers" were found that a definite probability became at once apparent. As the degree of carrier production was so high among troops who had never had any association with tropical areas, it seemed certain that the normal population of France and Great Britain must be an infective source. How the satisfactory examination has been achieved of sufficient civilians in this and other countries need not detain us. The task has been completed to a degree giving considerable finality to the observer's conclusions.

### The Percentage Incidence.

It is now certain that all the common intestinal protozoa (amœbæ and flagellates) of man occur in Britain at the present time. From a practical standpoint, however, the most important figures relate to the incidence of *E. histolytica* infection. "It has been demonstrated that not less than 3.4 per cent. of persons already examined harbour the parasite; and it is probable that this figure, which represents an absolute minimum, indicates that the true rate of infection is of the order 7 to 10 per cent." Not only this, but the dysentery amœba is almost certainly indigenous in Britain. Mr. Dobell admits that a large number of infected troops have returned, and that the extensive infection of civilians in general has been demonstrated only since the outbreak of war. The present situation might therefore be due to an infected soldiery, but this explanation is almost certainly incorrect. All his recent evidence is directly against it.

We know that long before the war *E. histolytica* occurred in British residents who had never left the country. We know the parasite occurs in widely separated parts of the country and is comparatively common. We know that it occurs at the present time in every part of the world where it has been sought; and from its relative harmlessness to human beings it seems probable that its association with mankind is no new thing. The very fact that *E. histolytica* is now comparatively common in persons who have never left the country, whilst amœbic diseases are uncommon, argues a degree of tolerance in the community such as is inconsistent with the supposition of its recent importation. Finally, it has not been possible . . . to establish any connection between native cases of infection and carriers returning from abroad.

At first sight this conclusion may appear to claim scientific interest alone, but the proved prevalence of these organisms offers a novel prospect for contemplation by the British physician. He will justifiably view these organisms with considerable suspicion. But what is their true hygienic importance? The author candidly states that at present an incomplete answer only can be given. Nevertheless, he puts forward a number of essential facts.

### The Importance to Home Medicine.

The most sinister meaning of these figures is that the inhabitants must frequently consume food and drink which have been exposed to gross contamination. Of all the protozoa considered, not one lives anywhere but in its human host, and the only possible conclusion is that in many ways our sanitation, though good, is not yet perfect. Another point the health services must remember is that the source of infection is not necessarily the returned soldier. Amœbic cysts may arise from hitherto unsuspected members of a family whose life and vocation give no clue, according to the old standards. The clinician will ask also whether these protozoa cause disease, and, if so, which of them. It has now been proved—not conjectured only—that two of them are facultatively pathogenic—namely, *Entamœba histolytica* and *Balantidium coli.* The former alone has been found indigenous in these islands. It produces active disease infrequently, but there is no longer any doubt that it is of the same species as the amœba of tropical dysentery. It is noteworthy that all foreign and British strains are equally and acutely pathogenic to the cat, while experience conclusively proves that abroad, as well as at home, the presence of the amœbæ in man does not necessarily result in acute dysentery.

The conditions in England are not, therefore, exceptional. As Mr. Dobell explains, they really illustrate a special instance of a general pheno-

**Amœbic Dysentery in Britain** (*continued*).

menon. The fact is, that apparently the protozoa occurring in Britain may have become suited to and tolerated by the Briton; similarly the protozoa of Africa and the African native. But if the Briton is infected in Africa the probability of acute disease is greatly magnified. In any case, we have to realise that in this country not only may this amœba be the cause of far more subacute disease than has ever been supposed, but also that, as a nation, we still have much to learn about individual and communal hygiene. Were we perfect in these respects transference, and therefore persistence, of an indigenous dysentery amœba would be utterly impossible. Even if it is not a frequent and deadly disease-producer in Britain, the history of *E. histolytica* should drive home a much-needed national lesson.

